# Differentially Expressed Circular RNA Profile in an Intracranial Aneurysm Group Compared with a Healthy Control Group

**DOI:** 10.1155/2021/8889569

**Published:** 2021-01-29

**Authors:** Yonggang Ma, Baoqing Zhang, Dong Zhang, Shuo Wang, Maogui Li, Jizong Zhao

**Affiliations:** ^1^Department of Neurosurgery, Beijing Tiantan Hospital, Capital Medical University, Beijing, China; ^2^China National Clinical Research Center for Neurological Diseases, Beijing, China; ^3^Center of Stroke, Beijing Institute for Brain Disorders, Beijing, China; ^4^Department of Neurosurgery, Weifang Yidu Central Hospital, 4138 Linglongshan Road, Qingzhou, Shandong Province, 262500, China

## Abstract

**Objective:**

Intracranial aneurysm (IA) is a fatal disease owing to vascular rupture and subarachnoid hemorrhage. Much attention has been given to circular RNAs (circRNAs) because they may be potential biomarkers for many diseases, but their mechanism in the formation of IA remains unknown.

**Methods:**

circRNA expression profile analysis of blood samples was conducted between patients with IA and controls. Overall, 235 differentially expressed circRNAs were confirmed between IA patients and the control group. The reliability of the microarray results was demonstrated by quantitative real-time polymerase chain reaction (qRT-PCR).

**Results:**

Of 235 differentially expressed genes, 150 were upregulated, while the other 85 were downregulated. Five miRNAs matched to every differential expression of circRNAs, and related MREs were predicted. We performed gene ontology (GO) analysis to identify the functions of their targeted genes, with the terms “Homophilic cell adhesion via plasma membrane adhesion molecules” and “Positive regulation of cellular process” showing the highest fold enrichment.

**Conclusions:**

This study demonstrated the role of circRNA expression profiling in the formation of IA and revealed that the mTOR pathway can be a latent therapeutic strategy for IA.

## 1. Introduction

Intracranial aneurysm (IA) is a disease characterized by abnormal dilatation of the intracranial artery as an abnormal bulge. Increasing numbers of asymptomatic unruptured IAs have been discovered recently using computed tomography (CT) or magnetic resonance imaging (MRI). It has been proposed that 3% to 5% of individuals harbor an IA [1]. The pathogenesis and etiology of IA are not yet clearly understood. The possible factors for IA formation include inflammation, hemodynamics, genetics, hormones, and the environment [2–4]. A high proportion of subarachnoid hemorrhage (SAH) is due to IA rupture due to its high mortality rate. Smoking and high blood pressure were considered to be risk factors for IA rupture [5]. Several studies have confirmed several loci contributing to IA formation and rupture [6–17]. Among them, 4 loci, including 1p34.3—p36.13, 7q11, 19q13.3, and Xp22, have been replicated in different studies [8, 10, 11, 18, 19]. Recent studies have observed that circular RNAs (circRNAs) are linked to cerebrovascular diseases such as moyamoya disease, atherosclerosis, and ischemic stroke. Nevertheless, the pathogenesis of IA formation and rupture remains poorly understood.

circRNAs are noncoding circular RNAs that are single stranded without free terminals [20]. They bond continuous loops by connecting their 3′- and 5′-ends and are abundant in eukaryotic cells [20]. In light of the literature, circRNAs are associated with the pathogenesis of a number of diseases, such as moyamoya disease and stroke. circRNAs can modulate the gene expression during transcriptional or posttranscriptional processes through three main pathways: acting as microRNA (miRNA) sponges, holding RNA binding proteins (RBPs), and controlling alternative splicing and parental gene expression [20, 21]. Previous research has suggested that circRNAs are deeply related in many vascular diseases [22]. For instance, circular antisense noncoding RNA is linked to atherosclerosis by regulating inhibitors of cellular factor expression [23]. Moreover, Zheng et al. found that one circRNA was highly expressed in aortic tissues of patients with aortic aneurysm compared with healthy tissue. However, the connection between circRNAs and IA is still unknown. To examine how circRNAs regulate the formation of IA and identify the potential molecules contributing to IA rupture, we carried out this study to analyze circRNA profiles among unruptured IA patients (UIA) and ruptured IA (RIA) patients and controls.

## 2. Materials and Method

### 2.1. Study Population and Blood Sample

From January to March 2019, we recruited 5 healthy people aged 20 to 60 years old. All 5 healthy controls had undergone computed tomography angiography (CTA) examination and healthy check-ups within half a year. Participants diagnosed with IA from January to March 2019 in our institute were also included in this study. All enrolled participants underwent CTA or digital subtraction angiography (DSA) examination. Furthermore, computed tomography (CT) plain scanning was conducted to evaluate whether IAs ruptured among all IA patients. Based on plain CT imaging, we divided the IA patients into two subgroups: unruptured IA patients and ruptured IA patients. Patients with other serious cardiovascular or cerebrovascular diseases were eliminated to avoid confounding results. Our research was permitted by the Ethics Committee Review Board of Beijing Tiantan Hospital. Informed consent was acquired from all enrolled participants before blood sample collection.

Blood samples were drawn from individuals and subsequently collected in vacuum collecting tubes. Samples were centrifuged at 1500 g/min for 15 minutes, and blood cells were removed. To remove cell debris, the clear supernatant was transferred into a new centrifuge tube (RNase-free) and centrifuged at 10000 g/min for 10 min. We transferred the supernatant into a new RNase-free centrifuge tube and stored it at -80°C immediately.

### 2.2. RNA Isolation and Purification

Five samples of normal peripheral blood, five samples of UIA patients' peripheral blood and five samples of UIA patients' peripheral blood were prepared for RNA extraction. The nurse drew a 5 ml volume of blood from each subject by venipuncture and collected them in K 2 EDTA-coated vacutainer tubes (BD Biosciences, Franklin Lakes, NJ, USA). Total RNA was extracted from the blood samples using TRIzol reagent (Invitrogen, Carlsbad, CA, USA) based on the manufacturer's protocols. A NanoDrop ND-1000 (Thermo Fisher Scientific, Wilmington, DE, USA) was used to quantify the total RNA. RNA integrity was checked by agarose gel electrophoresis. RNase R (Epicentre, Inc.) was used to discard linear RNAs and enrich circular RNAs.

### 2.3. RNA Labeling and Hybridization

RNA was amplified and labeled by the Arraystar Super RNA Labeling Kit (Arraystar) according to the manufacturer's protocols, and then, we purified the labeled cRNA using a RNeasy mini kit (Qiagen, Hilden, Germany). The concentration and specific activity of the labeled cRNAs (pmol Cy3/*μ*g cRNA) were determined by a NanoDrop ND-1000. Subsequently, the qualified labeled cRNA samples (yield > 1.65 *μ*g and specific activity > 9.0) were hybridized onto Arraystar Human circRNA Arrays (V2, Arraystar Inc.) Then, the microarray slides were incubated for 17 hours at 65°C in a hybridization oven (Agilent Technologies, Inc., Santa Clara, CA, USA). Hybridized arrays were washed, fixed, and scanned with a G2505C scanner (Agilent Technologies).

### 2.4. Data Analysis and Bioinformatics

Original data were fetched by Feature Extraction software v. 11.0.1.1 (Agilent Technologies). We performed quantile normalization of the raw data and subsequent data processing using the limma package in R v.3.3 software. After quantile normalization of the raw data, low-intensity filtering was conducted, and the circRNAs that had at least 5 out of 15 samples with flags in “Present” or “Marginal” (defined by GeneSpring software) were retained for further differential analysis.

When we compared the two groups of profile discrepancies (the IA group versus the control group), the “fold change” (i.e., the ratio of the group averages) between the groups for each circRNA was computed. The statistical significance of the difference was estimated by *t*-test. circRNAs with fold changes of ≥1.3 and *P* values of < 0.05 were selected as obviously differentially expressed.

Several studies have shown that circRNAs play a crucial role in regulating gene expression, which fine tunes the level of miRNA by sequestering miRNAs. Their reciprocity with diseases related to miRNAs suggests that circRNAs are crucial for disease occurrence. In our study, the circRNA/microRNA interactive relationship was predicted with Arraystar's homemade miRNA target prediction software based on TargetScan [24] and miRanda [25], and we annotated at length the differential expression circRNAs among all the groups based on circRNA/miRNA interactive information. All differentially expressed circRNAs were evaluated by GO (Gene Ontology) and KEGG (Kyoto Encyclopedia of Genes and Genomes) pathway analyses. To identify the target genes for the predicted miRNA sponge, we used the network visualization and analysis tool Cytoscape v.3.4 to generate ircRNA-miRNA networks. Furthermore, pathway network analysis was performed to evaluate reciprocal relationships among the pathways and visualize the network using Cytoscape v.3.4 software.

### 2.5. Quantitative Real-Time (qRT)-PCR

qRT-PCR was carried out to identify the microarray results. We synthesized cDNA from the total RNA using Super Script III reverse transcriptase (Invitrogen) based on the manufacturer's protocol. The PCR thermocycling conditions of predenaturation were set at 95°C for 10 minutes, followed by 40 cycles (95°C for 10 seconds and 60°C for 60 seconds). We assigned the housekeeping gene *β*-actin as the internal reference. All reactions were performed in triplicate, and the 2 ^−*ΔΔ*Ct^ method was used to calculate the expression level of circRNAs.

### 2.6. Statistical Analysis

Student's *t*-test (two tailed) and chi-squared test were used to assess the statistical significance between groups. Statistical analysis was conducted using SPSS 22.0 (SPSS Inc., Chicago, IL, USA). A *P* value of < 0.05 was considered statistically significant.

## 3. Results

### 3.1. Analysis of circRNA Expression Profiles

**Figure 1:**
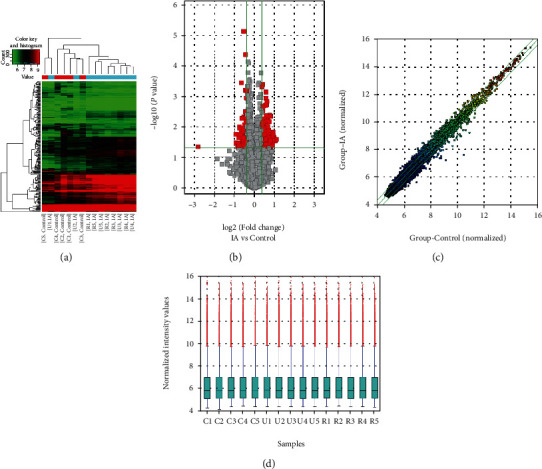
circRNA expression profile comparison among three groups. (a) Hierarchical clustering demonstrates a difference in circRNA expression profiling between the three groups. (b) Volcano plot shows differential circRNA expression between the IA patients and control groups, and the red points represent the circRNAs with log 2.0-fold changes (upregulated and downregulated) with statistical significance (*P* < 0.05). (c) Scattered plot shows the circRNA expression variation between the IA patients and control groups. (d) Box plot shows the distribution of circRNA expression patterns of three groups. IA: aneurysm.

We implemented microarray analysis of five samples from the UIA group, five from RIA patients, and five samples from matched normal controls. The general information and clinical baseline data of all participants are shown in Table 1. To prevent other probable risk factors from confounding the results, we matched the size, shape, and location of aneurysms between UIA patients and RIA patients. The expression of 12546 circRNAs was evaluated in the three groups by microarray analysis. The difference in circRNA expression profiles between the IA patients and healthy group and between the UIA group and RIA group were revealed by hierarchical clustering. Volcano, scatter, and box plots were generated to demonstrate the differentially expressed circRNAs (Figures 1(a)–1(d)). Of all differentially expressed circRNAs, 150 were upregulated, and 85 were downregulated between the two compared groups (IA group and control group). Seven upregulated and 3 downregulated differentially expressed circRNAs were validated between the UIA group and RIA group (fold difference of ≥1.3 and *P* value of < 0.05) [26].

### 3.2. qRT-PCR Validation of Differential Expression of circRNAs

**Figure 2:**
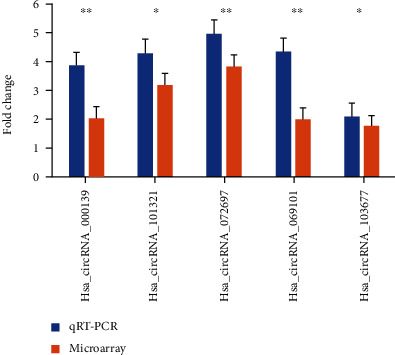
qRT-PCR validation of the differentially expressed circRNAs.

Five circRNAs (hsa_circRNA_000139, hsa_circRNA_101321, hsa_circRNA_072697, hsa_circRNA_069101, and hsa_circRNA_103677) were selected for qRT-PCR to confirm the microarray analysis data in fifteen IA and control samples. The qRT-PCR results were in accordance with the microarray expression data, indicating the high reliability of the microarray analysis (Figure 2).

### 3.3. ceRNA Network Construction and GO and KEGG Pathway Analyses

**Figure 3:**
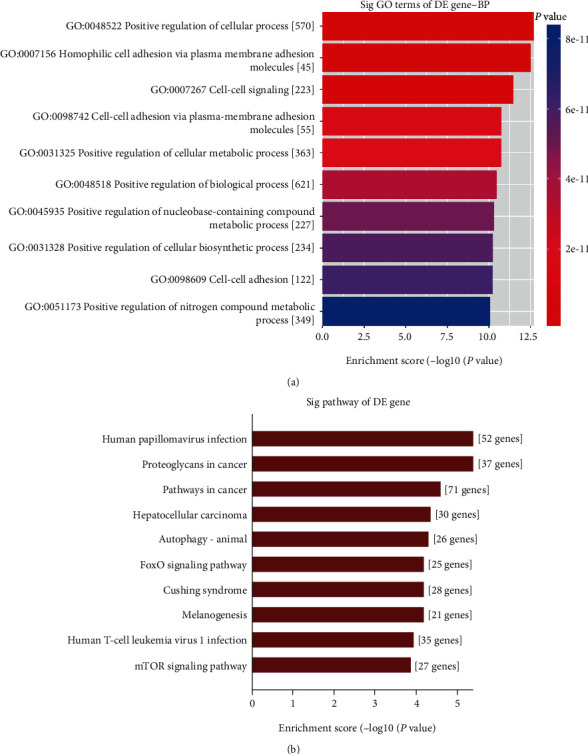
GO and KEGG pathway analyses of the differential expression circRNAs. (a), Top ten GO biological processes. (b), KEGG pathways of differential expression circRNAs. GO: Gene Ontology; KEGG: Kyoto Encyclopedia of Genes and Genomes.

**Figure 4:**
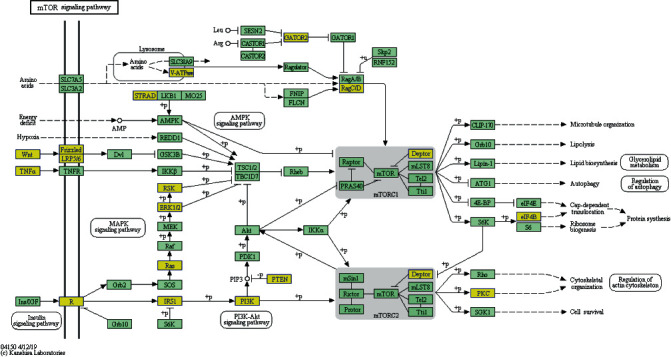
Target genes in the mTOR signaling pathway. mTOR: mammalian target of rapamycin.

circRNAs can modulate gene expression during transcriptional or posttranscriptional processes by functioning as inhibitors of miRNA bonding sponges or partners. We predicted that the five feasible miRNAs matched to every differentially expressed circRNA and MRE. Additionally, we evaluated 2465 circRNA-miRNA pairs with one or more binding sites. The interactive network between circRNAs and miRNAs was predicted for verified circRNAs, and GO analysis was carried out to identify the role of targeted genes. The top ten enriched GO terms in biological process are shown in Figure 3 with terms “Homophilic cell adhesion via plasma membrane adhesion molecules” and “Positive regulation of cellular process” showing the greatest fold enrichment. The KEGG pathway evaluation proclaimed 10 obviously enriched pathways in correspondence to the targeted genes. The most significant pathways included hepatocellular carcinoma, proteoglycans in cancer, and the mammalian target of rapamycin (mTOR) signaling pathway (Figure 4).

## 4. Discussion

The formation mechanism of IA is still unknown. Previous research has reported that inflammation and hemodynamic, genetic, hormonal, and environmental factors may contribute to IA [10–12]. In addition, several studies have confirmed that circRNAs are relevant to cerebrovascular diseases such as moyamoya disease, atherosclerosis, and ischemic stroke. In this study, we used a circRNA microarray to demonstrate 235 differentially expressed circRNAs in IA patients compared a matched control group.

Noncoding RNAs are crucial regulators of gene expression. Studies have revealed that they can act on different cellular processes and biological functions by controlling gene regulation [27]. Noncoding RNAs, including linear RNAs and circRNAs, could serve as competing endogenous RNAs (ceRNAs) by competitively binding miRNA response elements. circRNAs mainly originate from genes that code for protein synthesis and are generally composed of exons [28]. It has been reported that circRNAs can exist in every tissue and organ; however, they are also tissue enriched in some situations [28, 29]. circRNAs could not be decomposed by RNase R because their 3′- and 5′-ends form a circle. Their stable characteristics are attributed to this special structure. Furthermore, researchers have appealed that circRNAs are conserved between species, and the expression levels of circRNAs are commonly cell or tissue specific, suggesting that circRNAs could be disease markers. Previous studies have demonstrated the crucial role of circRNAs in the occurrence of various cardiovascular diseases by regulating cytodifferentiation, proliferation, necrosis, and apoptosis [30]. Several experts have harbored the idea that heart-related circRNA could prevent the heart from hypertrophy and failure [31]. It could bind with miR-223, which downregulates the level of apoptosis repressor [31]. It was previously described that Cdr1as could sequester miR-7 in neurocytes via sequence complementarity [32, 33]. Moreover, Cdr1as has been demonstrated to have the capacity to regulate myocardial infarction by binding to miR-7a in mouse cardiomyocytes [34]. Wang et al. described that circRNAs are indicative of the regulation of heart mitochondrial dynamics and myocardial cell apoptosis [30]. Liu et al. reported the relationship between circR-284 and cerebrovascular ischemic stroke associated with the carotid artery. It has been found that the levels of circR-284 were higher in patients with carotid-related ischemia than in a control group [35].

circRNAs are also linked to various diseases, including Alzheimer's disease. It has been shown that circRNA ciRS-7/CDR1as is associated with amyloid peptide clearance by regulating miRNA miR-7 [36, 37]. Additionally, circRNAs have been proven and proposed to be related to several kinds of tumors. One research group revealed that high expression of circ-100338 could promote the proliferation of hepatocellular carcinoma cells [38]. In another study, Pang et al. determined that circ_0072309 could retard the progression of NSCLC by inhibiting the expression of miR-580-3p [39].

In our experiments, we analyzed circRNA levels in whole blood samples using a circRNA microarray. circRNA profiling was performed using five samples from every group. In total, 235 differentially expressed circRNAs were verified; 150 were upregulated versus 85 were downregulated between the two IA groups and the control group, whereas 7 upregulated versus 3 downregulated differentially expressed circRNAs were confirmed between the UIA group and the RIA group. The results implied that circRNAs were associated with the formation of IA. The interaction network between circRNAs and miRNAs was predicted for verified circRNAs, and GO analysis was carried out to identify the role of their targeted genes. We demonstrated that the term “homophilic cell adhesion via plasma membrane adhesion molecules” was the top enriched term in the analysis. This provided overwhelming evidence for the involvement of circRNAs in the formation of IA because previous studies have described the correlation between IA and cell adhesion. Peters et al. discovered that SPARC was highly expressed in aneurysms and is a counteradhesive glycoprotein that is expressed in a variety of tissues [40]. mTOR signaling is at the core of the pathway-pathway interaction network and is linked to the genes associated with IA.

This pathway modulates translation initiation of protein synthesis, and biological processes, such as cell size and proliferation, are regulated by this pathway, which could coordinately integrate nutrient sufficiency signals from mTOR with growth factor signals [41]. Similarly, the phospho-mTOR levels were decreased in ruptured IAs compared to unruptured IAs [42].

## 5. Conclusion

In conclusion, our study predicted the function of circRNAs in IA formation and expanded the body of knowledge on the pathogenesis of IA. We demonstrated that circRNAs are linked to the formation of IA by modulating the mTOR signaling pathway. Our results revealed a list of potential diagnostic biomarkers for IA and suggested that differentially expressed circRNAs or the mTOR pathway can be latent therapeutic strategies for IA.

## Figures and Tables

**Table 1:** Clinical characteristics of all participants.

Variable	IA patients		Control	*P* value
	UIA	RIA		
Number	5	5	5	
Age	55.60 ± 8.562	53.80 ± 8.585	45.60 ± 6.309	0.113
Size	6.20 ± 1.304	9.00 ± 0.743	NA	0.153
Location of IA			NA	
MCA	3	3		
ACA	2	2		
Shape (saccular)	5	5	NA	
Smoking	0	0	0	
Drinking	0	0	0	

## Data Availability

The raw data used to support the findings of this study are available from the corresponding author upon request.

## Supplementary Materials

Supplementary Materials The supplementary materials include three files. The primer file lists the primer sequences, lengths, and target genes. The sample file shows the quality and concentration of the 15 perineal blood samples. In addition, the network file shows the circRNA-miRNA networks.
