# Supplementary material for: Differentially Expressed Circular RNA Profile in an Intracranial Aneurysm Group Compared with a Healthy Control Group
Source: Dis Markers. 2021 Jan 29;2021:8889569. doi: 10.1155/2021/8889569 (PMC7864737; doi:10.1155/2021/8889569)
Supplement: Supplementary Materials — The supplementary materials include three files. The primer file lists the primer sequences, lengths, and target genes. The sample file shows the quality and concentration of the 15 perineal blood samples. In addition, the network file shows the circRNA-miRNA networks. [file 8889569.f1.zip › primer.docx]

| **gene** | **primer sequence** | **annealing temperature (℃)** | **Length(bp)** |
| --- | --- | --- | --- |
| β-actin | F:5’GTGGCCGAGGACTTTGATTG3’  R:5’CCTGTAACAACGCATCTCATATT3’ | 60 | 73 |
| hsa_circRNA_000139 | F:5’TGTGAGGGAAAAGACCCAATA3’  R:5’ATCCAGCTTTTTCTTCTCAGTAAC3’ | 60 | 56 |
| hsa_circRNA_101321 | F:5’GAAGGGATTTCCTGTTCTTTCTAA3’  R:5’CACCAAAATTCAGCTCCTGTAAC3’ | 60 | 86 |
| hsa_circRNA_079265 | F:5’CCTTTTTTGTCCCCCAACTT3’  R:5’CACCATCACGCCTCATTTTTA3’ | 60 | 140 |
| hsa_circRNA_005054 | F:5’CTTTCAAGGACAAACTAGAGGAG3’  R:5’GTGATACCCTCAGAAAAGACAGA3’ | 60 | 123 |
| hsa_circRNA_069101 | F:5’TGGGATGGAAGTTATTTGGGAA3’  R:5’CCATTTGTAGAGGTGGAGAGTTTT3’ | 60 | 123 |
| hsa_circRNA_103677 | F:5’TCCCAGACAATTCTCGTTACTCC3’  R:5’CATTGAGGTCTGGCATGTTATTG3’ | 60 | 138 |
| hsa_circRNA_001714 | F:5’GATTATGAGGTGGCTGGTTTGC3’  R:5’GCTGCTTTGTGCTTCCGTATTC3’ | 60 | 76 |
| hsa_circRNA_061260 | F:5’TACCAACCAACGGCTGGAAAT3’  R:5’CAAAAGAACTGATCTAAGAGGTCTCC3’ | 60 | 90 |
| hsa_circRNA_072697 | F:5’CAGATTTGTAACTGGAAAACTCATG3’  R:5’GCACTGGGGAGATTATCAAGATAG3’ | 60 | 92 |
